# Effects of Geomaterial-Originated Fillers on Microstructure and Mechanical/Physical Properties of α- and β-Chitosan-Based Films

**DOI:** 10.3390/molecules26247514

**Published:** 2021-12-11

**Authors:** Abdellah Mourak, Mohamed Hajjaji, Abdelhakim Alagui, Patrick Martin, Nicolas Joly

**Affiliations:** 1 Laboratoire des Sciences des Matériaux et Optimisation des Procédés, Faculté des Sciences Semlalia, Université Cadi Ayyad, B.P. 2390, Av. Pce My Abdellah, Marrakech 40001, Morocco; abdellah.mourak@edu.uca.ac.ma (A.M.); alagui@ucam.ac.ma (A.A.); 2 Unité Transformations & Agroressources—ULR7519, UniLasalle, Univ. Artois, F-62408 Béthune, France; patrick.martin@univ-artois.fr (P.M.); nicolas.joly@univ-artois.fr (N.J.)

**Keywords:** chitosan-based films, geopolymer, montmorillonite, palygorskite, mechanical/physical properties, microstructural characterization

## Abstract

Edible films and coatings with good mechanical/physical properties are highly required for carrying medical substances and food packaging. So, solvent-cast films of α- or β-chitosan filled with palygorskite, montmorillonite or geopolymer-containing material (GCM), were prepared, and the effects of their clay contents (up to 50 wt.%) on the mechanical/physical properties were assessed. The microstructure of the films was investigated using FT-IR spectroscopy, SEM and thermal analysis. The results showed that, except for the films composed of GCM and β-chitosan, the mechanical properties of the films with limited (up to 5 wt.%) to moderate (5–25 wt.%) amounts of fillers increased as a result of the attractive electrostatic forces formed between the fillers and chitosan functional groups (–NH_3_^+^, CH_2_OH and NHCOCH_3_). However, due to the occurrence of coarse aggregates, the strength of filler-rich films declined. The addition of fillers led to an increase in porosity and water absorption of the films, but it had irregular effects on their wettability and water vapor transmission rate. These observations as well as the thermal stability of the films were discussed in relation to the characterization results.

## 1. Introduction

Conventional polymeric films are widely used for food packaging as they prevent foods from spoilage and extend their shelf-life. However, as petroleum-based products, these types of films resist biodegradation. The widespread distribution and accumulation of these products in the environment have become a matter of a great concern [1]. Therefore, much attention has been paid to the synthesis of eco-friendly films with good performance [2,3,4].

Chitosan, which is a chitin-derivative biopolymer, is a convenient natural material for the preparation of thin, edible, biodegradable and antibacterial films [5]. It is a copolymer composed of glucosamine and N-acetylglucosamine units, and it exists in three allomorphic forms: α, β and γ. As compared to the β form, the α-chitosan (α-chitin-derivative) is most abundant and consists of antiparallel chain orientation with strong inter- and intra-molecular bonds [6,7].

The incorporation of limited amounts of montmorillonite (chemical formula: 

M_x_(Al_4-x_,Mg_x_)_2_Si_8_O_20_(OH)_4_, M is a charge compensating cation, and 0.5 < x < 1.3) to chitosan-based films improves their barrier ability and mechanical properties [7]. These positive effects are essentially related to the outstanding characteristics of the montmorillonite: nano-lamellar structure, high aspect ratio and cation exchange capacity, and expandability of the interlayer space. Montmorillonite, as an anionic mineral species, is able to form bonds with the protonated aminogroups of the chitosan. In such a condition, tough particles/chitosan interfaces form [8], and the stress transfer across the montmorillonite-chitosan film is facilitated. So, the mechanical strength of the films is enhanced. 

The studies dealing with the physical/mechanical properties of chitosan-based films have been mostly performed on α-chitosan films containing one nano-dimension filler, e.g., montmorillonite. However, much less attention has been paid to the study of the β-chitosan films and to the effects of the incorporation of two or three nano-dimension fillers, such as palygorskite and geopolymer. In this respect, it should be noted that palygorskite is a natural hydrated magnesium aluminum silicate (ideal chemical formula: Si_8_Mg_5_O_20_(OH)_2_(H_2_O)_4_.4H_2_O). It is a fibrous mineral with a hollow brick-like structure, and the channel cross-section is of 3.7 Å × 6.4 Å [9]. Palygorskite and montmorillonite are naturally occurring non-harmful minerals. Both are used as pharmaceutical excipients and gastrointestinal protectors [10,11,12]. The geopolymer is a synthesized aluminosilicate material (kaolinite derivative) presenting a polymeric structure [13]. Because of its biocompatibility, high porosity and good mechanical strength, the geopolymer has been deemed suitable for bone restoration [14] and drug carrying [15].

The aim of this work was to study the effects of the additions of montmorillonite, palygorskite and geopolymer-containing material (GCM) on the performance of the α- and β-chitosan-based films. For these purposes, the main physical and mechanical properties of films were followed as function of filler additions, and the microstructure of the films was also investigated.

## 2. Results and Discussion 

### 2.1. Mechanical and Physical Properties of the Composite Films

**Figure 1:**
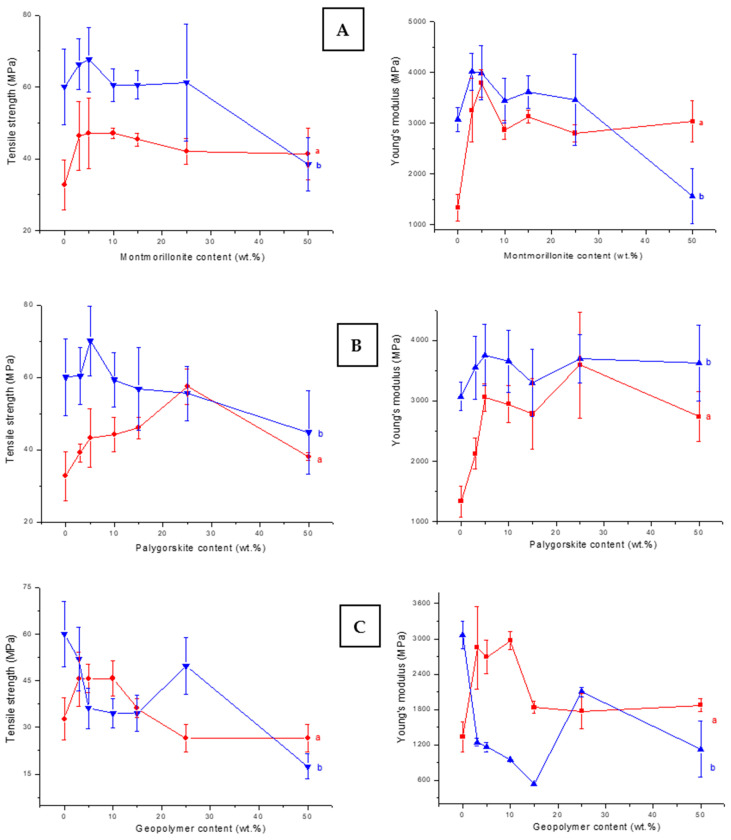
Variations in tensile strength and Young’s modulus of films prepared as a function of the fillers additions. (**a**) (α-chitosan)-based films. (**b**) (β-chitosan)-based films. (**A**) montmorillonite-, (**B**) palygorskite-, and (**C**) GCM-containing films.

Taking as a reference the mechanical properties of pure α-chitosan film, the tensile strength as well as the Young’s modulus of the composite films were enhanced with limited additions of montmorillonite or palygorskite (Figure 1A,B). The maximum increments of tensile strength (45 and 75% for montmorillonite- and palygorskite-containing films) were achieved with 5 wt.% montmorillonite and 25 wt.% palygorskite. In these conditions, Young’s modulus drastically increased (>150%). The blend of α-chitosan with small to moderate amount of GCM also improved the mechanical resistance of α-chitosan-based films (Figure 1C). The mechanical properties of the latter films reached their maximum in the range of 4–10 wt.% GCM. The improvement of the α-chitosan-based films’ mechanical properties was accompanied by a decrease in the elongation at break (from 20 to 40%). A positive increase in the elongation at break was measured for β-chitosan-based films, particularly those composed of GCM (maximum increment of the elongation at break: 225%). In this respect, it could be noted that the elongation at break of pure α- and β-chitosans films was 2.5% and 1.9%, respectively.

As can be deduced from Figure 1, the tensile strength of β-chitosan-based films composed of 5 wt.% montmorillonite or palygorskite was enhanced by ~14%. In parallel, the Young’s modulus of montmorillonite- and palygorskite-containing films increased by about 30% and 23%, respectively. In contrast, the mechanical properties of films composed of the β-chitosan and GCM declined.

As the mechanical properties of the polymer matrix composites are influenced by the interactions between the constituents and the filler particle distribution, among others [16], it was believed that in the presence of limited amounts of the fillers studied, chitosan chains and filler particles developed strong attractive forces, except for GCM-β chitosan composites. The formation of tough interfaces of chitosan/filler made easy the load transfer across the film [8]. So, the strength was evenly distributed, and therefore the mechanical resistance of the composites was improved. In contrast, the use of a high amount of the filler led to the formation of aggregates and heterogeneous zones throughout the film. Hence, the mechanical resistance dropped. Discussion regarding these assumptions is given in the characterization section.

**Figure 2:**
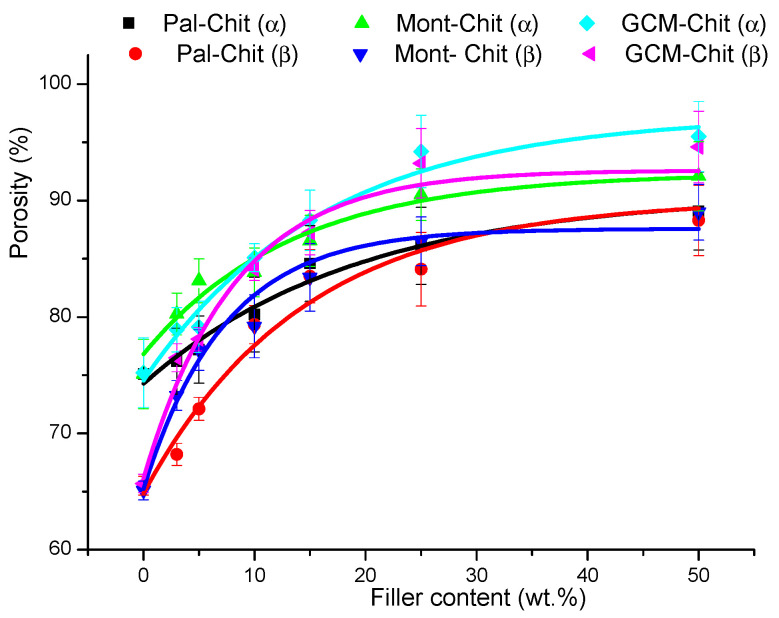
Changes in the porosity of the films studied versus the filler additions. Chit: chitosan; Pal: palygorskite; Mont: montmorillonite; GCM: geopolymer-containing material.

The addition of fillers to chitosans led to an increase in composite film porosity, and the curves obtained (Figure 2) fitted well with the equation:

(1) $$\ln\left(\frac{P_{0}^{max}-P_{o}}{A}\right)=-\frac{\tau_{f}}{k}$$

*τ_f_* is the filler content, $P_{0}^{max}$ is the maximum of porosity, and *A* and *k* vary in the ranges of 16–27 and 5–14, respectively.

The increase in film porosity depended on the nature of the filler in the following order: GCM>montmorillonite>palygorskite. The porosity of the films was different with respect to the filler type, and it was slightly higher for α-chitosan–based films. Based on these results, it emerged that the use of porous fillers contributed to the increase in chitosan-based film porosity.

**Figure 3:**
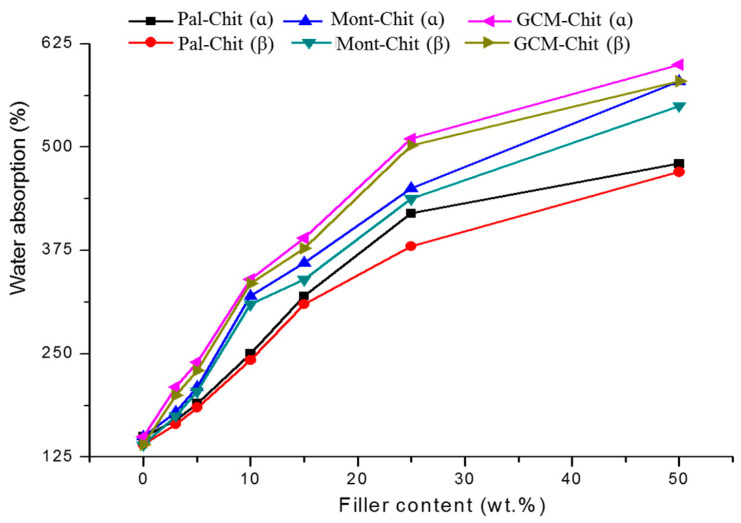
Variations in the amount of water absorbed by the composite films studied as a function of the additive contents. Chit: chitosan; Pal: palygorskite; Mont: montmorillonite; GCM: geopolymer-containing material.

The amount of water absorbed (*WA)* by the films increased with the increase in filler content (Figure 3), and the variation in *WA* versus *τ_f_* (previously defined) fitted well with the equation:

(2) *(WA)_max_* − *WA = Bexp (*−*τ_f_/b)*

*(WA)_max_* is the maximum amount absorbed, and *B* and *b* values were in the ranges of 400–530 and 20–29, respectively. 

It was thought that the water absorption was influenced by the porosity, which in its turn was dependent on the filler content. In fact, taking into consideration Equations (1) and (2), the water absorption was related to the porosity according to the equation:

(3) $$WA=C-D{\left(E-P_{o}\right)}^{\frac{k}{b}}$$

*C*, *D* and *E* are constant ($C={\left(WA\right)}^{max}$, D=BAkb and $E=P_{o}^{max}$).

**Figure 4:**
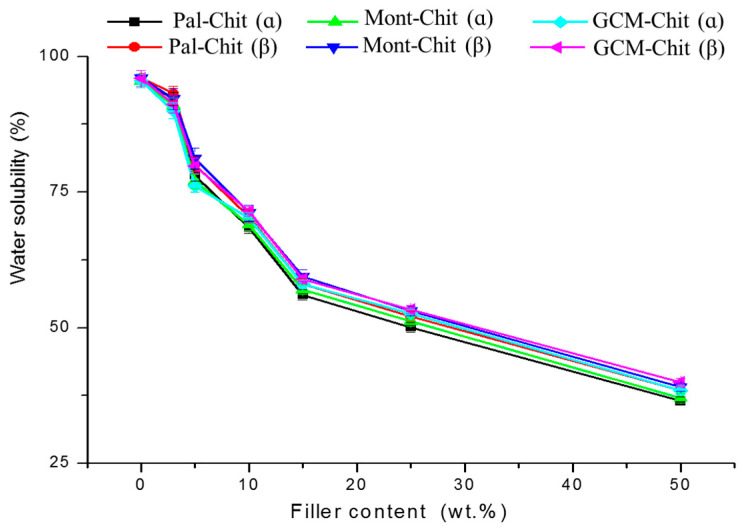
Evolutions of water solubility of the films against filler content. Chit: chitosan; Pal: palygorskite; Mont: montmorillonite; GCM: geopolymer-containing material.

The water solubility (*WS*) of the films decreased with increasing filler content (Figure 4), and the amount released was almost independent of the nature of the filler and the chitosan used. The change in *WS* against the filler content fit well with the relation:

(4) WS=(36±1)+(61±2)e−τf(17±1)

The solubility of the films was associated with the chitosan dissolution because the solution was subjected to the formation of a gel. The unexpected dissolution of chitosan was seemingly attributed to the decrease in pH of distilled water. The increase in water acidity was attributed to the quantitative dissolution of atmospheric carbon dioxide as the soaking tests were conducted for 24 h in open atmosphere. On the other hand, the evolution of *WS* curves allowed the deduction that the film stability in the operating conditions increased with the increase in filler amounts. It was believed that in such a condition, abundant and strong bonds formed between chitosan backbone and filler particles, and consequently, the chitosan was the object of restricted solubility.

**Figure 5:**
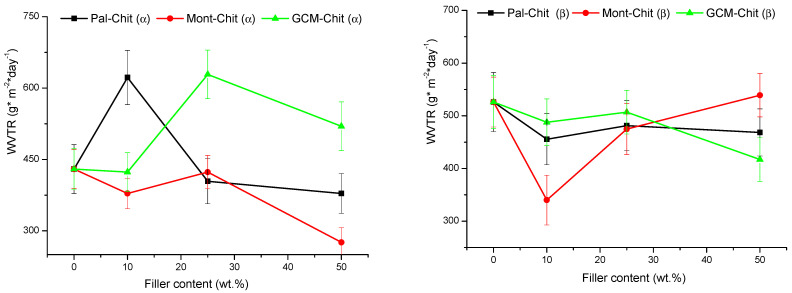
Changes in the water vapor transmission rate of the films investigated as a function of the filler additions. Chit: chitosan; Pal: palygorskite; Mont: montmorillonite; GCM: geopolymer-containing material.

The rate of water vapor transmission (*WVTR*) through montmorillonite- α-chitosan films decreased when montmorillonite content was <~5 wt.% or >25 wt.% (Figure 5). The *WVTR* decrease was estimated to be 12% and 35% for 5 and 50 wt.% for montmorillonite-chitosan films, respectively. In line with one author’s argument [17], it was believed that *WVTR* decreased because the diffusion phenomenon easily took place along the lengthy paths adjacent to the montmorillonite particles, which had large aspect ratios. On the other hand, the palygorskite additions (up to about 20 wt.%) to α-chitosan caused an increase in the *WVTR* (Figure 5) of the composite films, and the maximum transmission rate (~45%) was reached by 5 wt.%. In this case, the water vapor diffusion seemed to occur via the fibrous particles of palygorskite. However, in the presence of high amounts of palygorskite, this process appeared to be insignificant because the *WVTR* was almost similar to that of pure chitosan film. The additions of GCM as low as 10 wt.% did not affect the *WVTR* of the corresponding α-chitosan-based films (Figure 5); probably the geopolymer behaved like chitosan regarding water vapor transmission because of its polymeric structure and the homogeneous distribution of its particles. However, when the GCM amount exceeded 10 wt.%, the particle dispersion seemed to be irregular, and consequently the *WVTR* increased. Referring once again to Figure 5, the filler additions <25 wt.% to β-chitosan led to the decrease in the corresponding composite film *WVTR*, and the most significant reduction was obtained with the montmorillonite addition. This result supported the above comment that the *WVTR* was essentially related to the lengths of the paths in close proximity to the filler particles.

The changes in the wettability of the studied composite film surfaces versus filler content did not display regular evolutions, except for GCM-containing β-chitosan films. The wettability fluctuations (contact angles: 90–120 degrees) could be related to different wetting mechanisms possible, as the contact angle depends on surface roughness, surface energy and surficial functional groups (chemical heterogeneity), among others [18,19]. With some exceptions, the dewetting maxima were obtained with less than 5.5 wt.% filler, and the wettability reached its limit in the 10–15 wt.% range.

**Figure 6:**
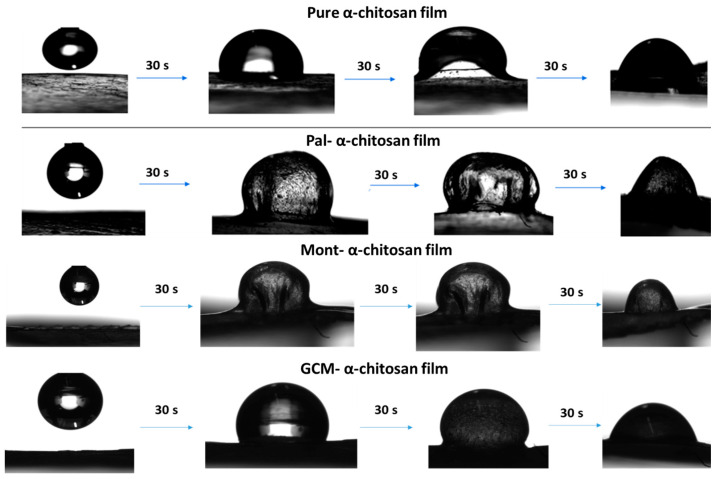
Typical micrographs showing instantaneous changes in droplet shape, and the local swelling of the pure chitosan and the composite films.

It is worth noting that in contact with the water droplet, films displayed a marked protuberance due to local swelling. This effect was attenuated as the droplet spread. Typical local swelling of the films and changes in droplet shape are shown in Figure 6.

Considering the results given above and referring to the common properties of edible films and common plastics [20,21], the studied films showed good tensile properties. However, they exhibited low elongation at break and resistance to water vapor transmission. Taking into consideration the reported studies related to this topic [22,23,24,25], we believed that these properties could be improved by incorporating adequate plasticizers and/or by chemical modifications of the chitosan and the filler particle surfaces.

### 2.2. Microstructural Characterization of the Films

**Figure 7:**
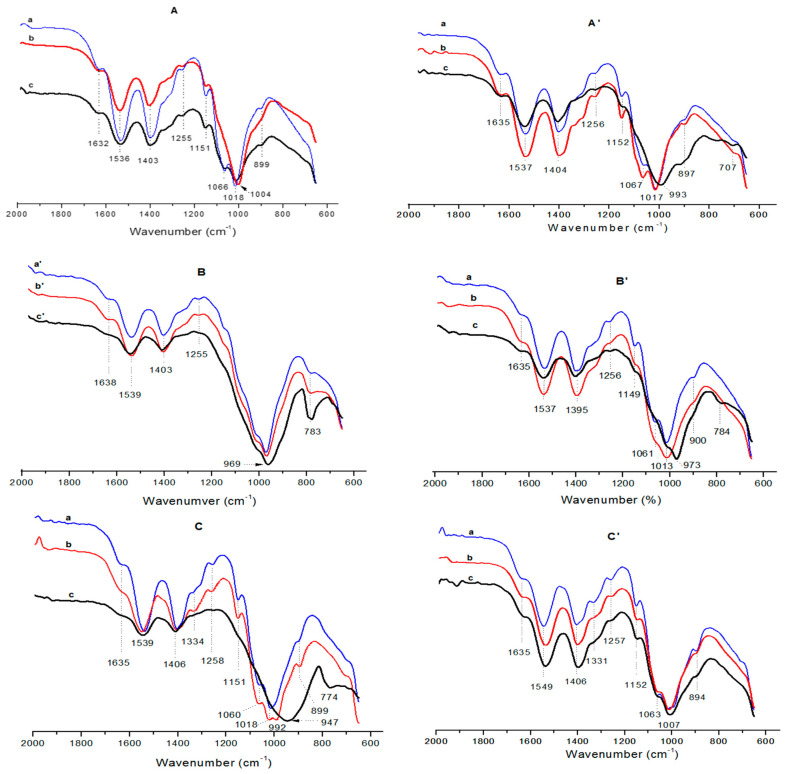
FT-IR spectra of the composite films studied.(**A**) montmorillonite-(α-chitosan); (**A’**) montmorillonite-(β-chitosan); (**B**) palygorskite-(α-chitosan); (**B’**) palygorskite-(β-chitosan); (**C**) GCM-(α-chitosan); (**C’**) GCM-(β-chitosan).(**a**) 3 wt.%, (**b**) 5 wt.%, (**c**) 25 wt.%, (**a’**) 15 wt.%, (**b’**)= **c**, (**c’**) 50 wt.%.

The comparative examination of the FT-IR spectra of pure α-chitosan film and corresponding composite films shown in Figure 7A–C exhibited a shift to lower frequencies for the vibrational bands associated with some bonds of the chitosan (Table 1), mainly due to the filler additions. The most significant shifting was noted for the protonated amine (–NH_3_^+^), the CH_2_ (CH_2_OH) and the CO (amide I, the primary and the secondary OH groups) bonds. In fact, as anionic species, montmorillonite and palygorskite are able to form attractive forces with –NH_3_^+^ groups, CH_2_OH and NHCOCH_3_ moieties of the chitosan. Similar electrostatic interactions should be formed between these functional groups and the geopolymer. Coulomb’s force and hydrogen bonding, which occurred between α-chitosan and filler particles, allowed reinforcement of the chitosan/particle interfaces. This result supported the above assumption that the strengthening of the films composed of limited amounts of fillers was essentially due to the formation of tough interfaces. Considering once again the FT-IR results (Table 1), the electrostatic attractive forces should still take effect in the presence of quantitative amounts of the fillers. So, the film weakening observed in this case should not occur. Thus, it was assumed that the mechanical strength of the filler-rich films was more impacted by the dispersion and piling up of the filler particles. 

Considering the FT-IR spectra of pure β-chitosan and β-chitosan-based composite films shown in Figure 7A’–C’ and the band assignments given in Table 2, the vibrational band (1537 or 1549 cm^−1^) related to the –NH_3_^+^ groups only appeared in the spectra of the composite films. In addition, the frequencies of the bands associated with the vibrations of CH_2_ and CO bonds were shifted. As previously mentioned, these facts were linked to the formation of electrostatic forces between β-chitosan and filler particles. So, the mechanical strength of composite films containing limited amounts of montmorillonite or palygorskite was enhanced (Figure 1). However, the effect of attractive electrostatic forces on the strength of GCM-containing films was less significant. Recalling the FT-IR results given in Table 2, the spectra of GCM-β chitosan films consisted of the band at 1331 cm^−1^ related to the bending vibration of C–H bonds of the chitosan pyranose unit ring. Thus, it was probable that there was no effective bond between β-chitosan chains and geopolymer particles.

**Figure 8:**
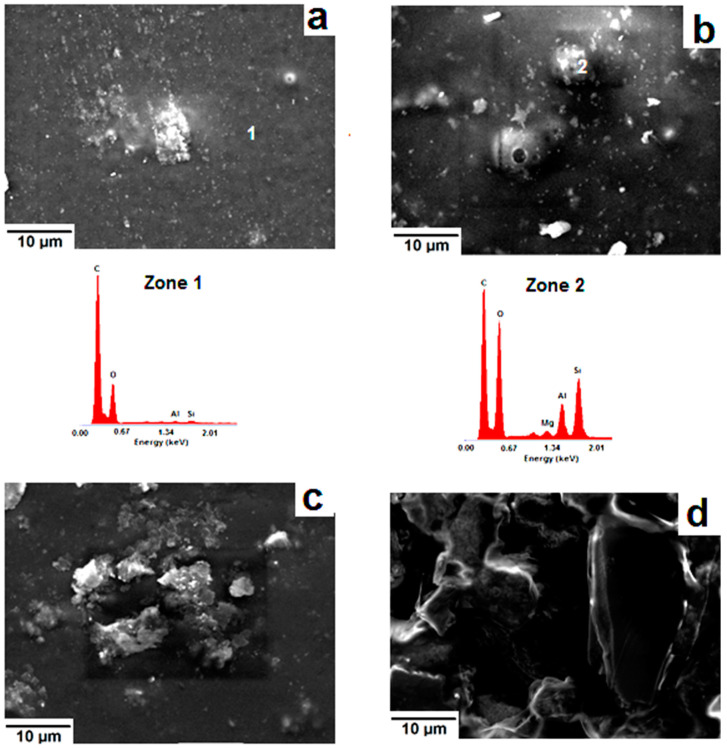
SEM micrographs of the montmorillonite-containing chitosan (α/β). (**a**,**b**) α-based films (**a**) 5 wt.%, (**b**) 25 wt.%); (**c**,**d**): β-based films (**c**) 5 wt.%, (**d**) 25 wt.%. Zone 1: EDS spectrum of the (α-chitosan)-rich zone; Zone 2: EDS spectrum of the embedded montmorillonite aggregate.

The SEM examination of α-chitosan-based film containing 5 wt.% montmorillonite exhibited an almost smooth surface with abundant fine embedded particles, identified as the aluminosilicate mineral used (Figure 8a). Conversely, coarse aggregates together with frequent bumpy and porous zones were seen in the montmorillonite-rich films (Figure 8b). The presence of these defects was responsible for the decline in mechanical properties and for the increase in porosity. Regarding the β-chitosan-based film containing 5 wt.% montmorillonite, aggregates such as seen in Figure 8c were formed. The occurrence of such aggregates did not have a negative impact on tensile strength or Young’s modulus of this film (Figure 1). Further addition of montmorillonite to β-chitosan led to a segregated microstructure (Figure 8d), and to a drop in mechanical resistance.

**Figure 9:**
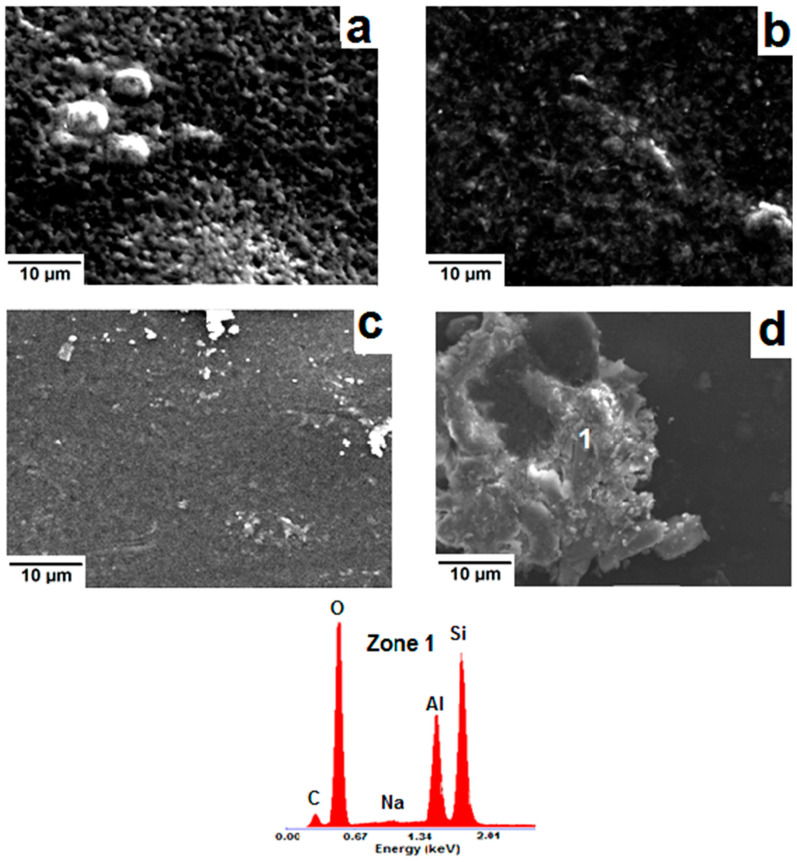
SEM micrographs of the α-chitosan (**a**) and the β-chitosan (**b**) films composed of 25 wt.% palygorskite. (**c**,**d**) are SEM micrographs of the α-chitosan and the β-chitosan films containing 3 wt.% GCM. Zone 1: EDS spectrum of the coarse aggregate shown in the micrograph (**d**).

The microscopic investigation of palygorskite-containing α-chitosan films, which manifested high mechanical performance, revealed an ordered pattern such as shown in Figure 9a. This framework seemed to be built of wrapped palygorskite fibers. On the other hand, numerous uncoated fibers of the palygorskite were found in β-chitosan-based film. A typical micrograph showing the microstructure of this film is presented in Figure 9b.

Because of the tiny size of the geopolymer grains and of their apparent ability to be mixed with α-chitosan, the GCM-(α-chitosan) films displayed a somewhat homogenous microstructure (Figure 9c). So, the film acquired good mechanical strength. This was not the case with the β-chitosan-based films, as coarse aggregates (Figure 9d) were heterogeneously dispersed across the matrix.

**Figure 10:**
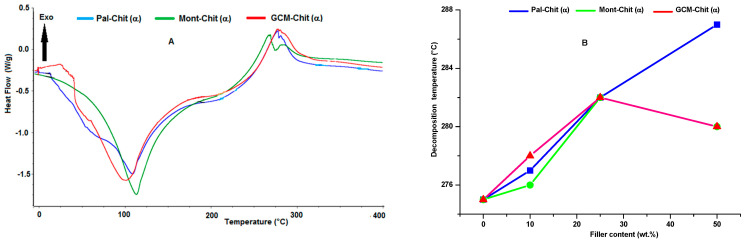
Thermograms of the α-chitosan-based films containing 10 wt.% of the fillers studied (**A**), and changes in the decomposition temperature of the α-chitosan-based films as a function of the filler contents (**B**). Chit: chitosan; Pal: palygorskite; Mont: montmorillonite; GCM: geopolymer-containing material.

As can be deduced from the typical thermograms given in Figure 10A, the thermal curves of studied films exhibited endothermic and exothermic effects in the 78–107 °C and 265–287 °C ranges, respectively. These thermal phenomena were ascribed to the loss of physisorbed water and to the decomposition of chitosan, respectively. The split of the exotherm, which was particularly observed in the montmorillonite-α-chitosan film thermal analysis, was assigned to the decomposition of chitosan of the matrix and to that located at the interlayer space of montmorillonite. Changes in the decomposition temperature of α-chitosan films as a function of the filler amounts displayed a linear ascendant evolution for palygorskite (Figure 10B). Similar evolution was observed in the case of montmorillonite addition to β-chitosan. Hence, the thermal stability differed according to the filler and chitosan characteristics. Based on data in the literature related to the thermal stability of clay-polymer nanocomposites [26], the improvement in film thermal decomposition was thought to be ascribed to the large aspect ratio of the additives and to the high interfacial area, among other factors. In such a condition, the barrier effect, which is a physical effect, is the phenomenon predominantly responsible for the enhancement of decomposition temperature. However, given the discrepancies between thermal behaviors observed in this study, the effect of chitosan’s inherent characteristics should be taken into consideration. As a biopolymer with parallel chains, weak intermolecular bonds and high molecular weight, β-chitosan mixed with montmorillonite gave rise to a fairly thermal resistant film. Concerning α-chitosan, which consists of antiparallel chains and has relatively low molecular weight, the improvement in thermal decomposition was obtained with the introduction of palygorskite.

## 3. Materials and Methods

### 3.1. Fillers

**Figure 11:**
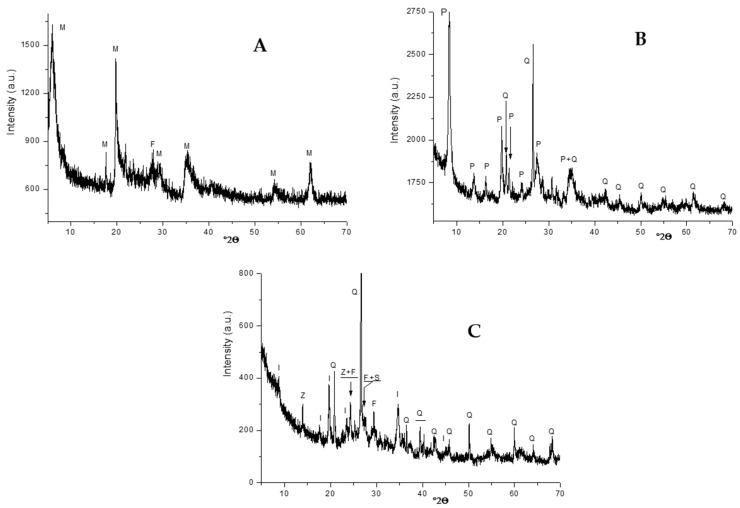
X-ray diffraction traces of the fillers used. (**A**) Montmorillonite-rich clay; (**B**) Palygorskite-rich clay; (**C**) Geopolymer-containing material. M: montmorillonite (PDF# 03-0010), F: feldspar (PDF# 76–0831), P: palygorskite (21-0958), Q: quartz (PDF# 05–0490), I: illite (PDF# 43–0685), Z: zeolite (PDF# 84–0698).

Local montmorillonite- and palygorskite-rich clays and geopolymer-containing material were used as additives. Typical X-ray diffraction patterns of these materials are shown in Figure 11.

Montmorillonite and palygorskite-rich clays were mildly ground, sieved (<100 μm) and decarbonated with a solution of acetic acid and sodium acetate (pH = 4.5). The treated clays were sodium-loaded using an NaCl solution (0.1 M), then rinsed and stored at 120 °C.

The geopolymer-containing material was an illitic-kaolinitic clay derivative. The starting clay (particle size <100 μm) was heated at 700 °C for 2 h to transform the kaolinite into metakaolinite, which is known as a suitable feedstock material for geopolymer synthesis [27]. The heated clay was etched with an NaOH solution (6 M) and cured at 83 °C for 30 days. The latter operating conditions were adopted based on the study by El Hafid and Hajjaji [28]. The cured material was abundantly washed with distilled water and oven-dried at 120 °C.

### 3.2. Obtention of α- and β-Chitosans

α- and β-chitosans were prepared by deacetylation of α- and β-chitins, which were extracted from local shrimp shells and squid pens, respectively. To extract chitins, the dried shells and pens were ground and subjected to demineralization and deproteinization treatments by using HCl solution (0.55 N) and NaOH solution (0.3 N), respectively. The obtained chitins were deacetylated with concentrated and hot solutions of NaOH (40 wt. % and 50 wt. % for α- and β-chitins, respectively). The solution temperatures and the etching durations were 120 °C and 24 h for α-chitin, and 80 °C and 12 h for β-chitin, respectively. The deacetylation of α-chitin was performed twice, while that of β-chitin was performed three times. More details regarding the preparation of α- and β-chitosans are given elsewhere [29].

Deacetylation degree (DD) of chitosans, determined following the procedures described by Brugnerotto et al. [30] and Tolaimate et al. [31], was found to be 93.5 ± 1.5%. The molecular weight (MW), which was measured following the method published by Kumar [32], was 23,000 and 883,650 g/mol for the α- and β-chitosans, respectively.

### 3.3. Film Preparation

An aqueous solution of 1% acetic acid (25 mL) and chitosan (1 g), and a dispersion (25 mL) of sodium-saturated filler (up to 1 g) were prepared and stirred separately for 3 h at ambient temperature. The chitosan limpid solution and the filler dispersion were then mixed together and stirred for 5 h. An additional stirring period (30 min) was applied using an ultrasonic bath. The mixture was casted in a glass Petri dish, and left at room temperature until total evaporation of water. The formed film was immerged in a 0.5 M NaOH solution, washed with distilled water and stored at 25 °C.

### 3.4. Measurements of Mechanical and Physical Properties

Measurements of tensile strength and Young’s modulus were conducted at room temperature on dumbbell-shape samples cut from the films. Mechanical tests were performed using an Instron 4466 apparatus (gauge length: 50 mm, width: 5 mm) functioning at across head speed of 10 mm·min^−^^1^.

The wettability of the films was evaluated at room temperature (around 25 °C). The water drop contact angle measurement was performed with the sessile-drop technique using an Apollo Instruments OCA 20 contact angle analyzer equipped with a video camera (water drop: 3 µL, time: 10 s). Measurements were obtained on both sides of the droplet by the ellipse-fitting calculation method.

Determination of the instantaneous water absorption (*WA*) was carried out on 1 cm × 1 cm pieces of the dried films. The pieces were weighed, soaked in 15 mL aqueous solution (0.9% NaCl) at 25 °C, and drawn out at regular times in order to be re-weighed. The *WA* corresponds to the weights’ difference to the initial weight of the sample.

Water vapor transmission rate (*WVTR*) of the films was determined following the experimental protocol detailed in ASTM E96-95, and by adopting the equation:

*WVTR* = 24·Δ*m/t·A* (5)

where *Δm* is the water weight change (g), t is the span (day) associated with the weight change, and *A* (m^2^) is the surface of the film used.

Water solubility (*WS*) measurements were performed on 4 cm × 4 cm samples cut from the films dried at 105 °C. The sample was weighed and immersed in distilled water (50 mL) at room temperature. After 24 h of soaking, the samples was drawn out and weighed. The water solubility was defined as the ratio of the weights’ difference to the weight of the dried sample.

*WS (%)* = *(M_i_ − M_f_)/M_i_* (6)

where *M_i_* is the initial mass and *M_f_* is the final mass of the sample.

Film porosity (*P_o_*) was calculated according to the relation:

*P_o_* = 1 − *ρ_a_/ρ_b_* (7)

*ρ_a_* and *ρ_b_* are respectively the apparent and true densities measured by the liquid displacement method.

### 3.5. Characterization Techniques

X-ray diffraction (XRD) analysis of chitosan-based materials was carried out on powdered samples using a Philips X’Pert MPD diffractometer operating with a copper anode (λ_Kα_ = 1.5418 Å). 

The Fourier transform-infrared (FT-IR) analysis of the films was performed with an Agilent Cary 630 FT-IR spectrophotometerfunctioning in the range of 4000–600 cm^−^^1^. The resolution was 5 cm^−^^1^.

The microscopic examinations of the films were carried out with a Zeiss SupraVP40 scanning electron microscope, equipped with a 20 mm^2^ X-Max diffusion silicon detector. To facilitate electron conduction, the samples were coated with a thin layer of carbon.

The thermal analysis of the films was performed using a Perkin-Elmer Diamond apparatus, operating under N_2_ atmosphere and a heating rate of 10 °C/min.

## 4. Conclusions

The strengthening of palygorskite-added α-chitosan solvent-cast films was essentially related to the typical patterned structure of the films and the attractive electrostatic forces formed with the chitosan. In spite of their different microstructures, α-chitosan films containing montmorillonite on one hand, and the GCM on the other hand, showed almost identical tensile strengths. Except for the GCM-containing films, β-chitosan-based films manifested lower mechanical resistance.

Film porosity was influenced by the filler nature and content, and the impact of the filler on the porosity followed the order: GCM > montmorillonite > palygorskite. The porosity had a marked influence on water absorption, but its impact on the water vapor transmission rate was not obvious. Relatively low values for the *WVTR* were obtained using chitosan films containing montmorillonite.

The studied films were somewhat hydrophobic, and their wettability changed irregularly with the filler additions, presumably because the contact angle depends much more on surface characteristics.

Compared to edible films and common plastics, the studied films showed good tensile properties, but they were somewhat stiff and less permeable to water vapor. Hence, we believe that the incorporation of plasticizers and/or chemical modification of chitosan as well as filler will allow the development of films that meet the required standard specifications.

## Figures and Tables

**Table 1:** Frequencies (cm^−1^) and assignments of FT-IR bands of pure and filler-containing chitosan-based film spectra.

α-Chitosan	Mont-α Chitosan			Pal-α Chitosan			GCM-α Chitosan			Assignment
	Montmorillonite Content (wt.%)			Palygorskite Content (wt.%)			GCM Content (wt.%)			
	3	5	25	15	25	50	3	5	25	
1658	1632	1632	1632	1638	1640	1639	1635	1635	-	νCO (amide I)
1598	1536	1536	1536	1539	1541	1538	1539	1547	1547	δ-NH_3_^+^
1428	1403	1403	1403	1403	1406	1411	1406	1406	1414	δ CH_2_ (CH_2_OH)
1383										δ CH_3_ (NHCOCH_3_)
1335							1334	1332	-	δ CH (pyranose ring)
1255	1255	1255	1255	1255	1250	-	1258	1261	-	NHCO group (amide III)
1151	1151	1151	1151				1151	1151	-	νCOC (glycosidic linkage)
1094	1066	-	1066				1060	1063	-	νCO (secondary OH group)
1038	1018	1004	1012				1018	1023	-	νCO (primary OH group)
							-	992	-	Geopolymer
				969	969	964				Palygorskite
							-	-	947	Geopolymer
899	899	-	893				899	896	-	pyranose ring
				783	786	780				Palygorskite
							-	-	774	Quartz
661										δNH out of plane
602										δOH out of plane

**Table 2:** Frequencies (cm^−1^) and assignments of FT-IR bands of the spectra of pure and filler-containing β-chitosan-based films.

β-Chitosan	Mont-β Chitosan			Pal-β Chitosan			GCM-β Chitosan			Assignment
	Montmorillonite Content (wt.%)			Palygorskite Content (wt.%)			GCMContent (wt.%)			
	3	5	25	3	5	25	3	5	25	
1621	1635	1635	1635	1635	1635	1635	1635	1635	1635	νCO (amide I)
	1537	1537	1537	1537	1537	1537	1549	1539	1537	δ-NH_3_^+^
1431	1404	1404	1404	1395	1395	1395	1406	1400	1400	δ CH_2_ (CH_2_OH)
1388										δ CH_3_ (NHCOCH_3_)
1323							1331	1331	1331	δ CH (pyranose ring)
1261	1256	1256	1256	1256	1256	1256	1257	1257	1257	NHCO group (amide III)
1111	1152	1152	1152	1149	1149	1149	1152	1152	1152	νCOC (glycosidiclinkage)
	1067	1067	-	1061	1061	1061	1063	1063	1063	νCO (secondary OH group)
1038	1017	1017	993	1013	1013	1013	1007	1007	1007	νCO (primary OH group)
				-	-	973				Palygorskite
888	897	897	897	900	900	-	894	894	894	pyranose ring
				-	-	784				Quartz
661	699	699	707							δNH out of plane
610										δOH out of plane

## Data Availability

The data used to support the findings of this study are available fromthe corresponding author upon request.

## Sample Availability

The studied samples are available from the authors.
